# Self-Attractive Semiflexible Polymers under an External Force Field

**DOI:** 10.3390/polym14214762

**Published:** 2022-11-07

**Authors:** Antonio Lamura

**Affiliations:** Istituto Applicazioni Calcolo, Consiglio Nazionale delle Ricerche (CNR), Via Amendola 122/D, 70126 Bari, Italy; antonio.lamura@cnr.it

**Keywords:** mesoscale simulations, nonequilibrium simulations, polymer dynamics

## Abstract

The dynamical response of a tethered semiflexible polymer with self-attractive interactions and subjected to an external force field is numerically investigated by varying stiffness and self-interaction strength. The chain is confined in two spatial dimensions and placed in contact with a heat bath described by the Brownian multi-particle collision method. For strong self-attraction the equilibrium conformations range from compact structures to double-stranded chains, and to rods when increasing the stiffness. Under the external field at small rigidities, the initial close-packed chain is continuously unwound by the force before being completely elongated. For double-stranded conformations the transition from the folded state to the open one is sharp being steeper for larger stiffnesses. The discontinuity in the transition appears in the force-extension relation, as well as in the probability distribution function of the gyration radius. The relative deformation with respect to the equilibrium case along the direction normal to the force is found to decay as the inverse of the applied force.

## 1. Introduction

The study of single polymers, such as, for example, DNA, filamentous actin, and microtubules under various flow conditions, has helped in understanding their dynamical and conformational properties [1]. The first investigations of the flow behavior of single DNA filaments [2] opened the way to a large variety of flow experiments which provided insight into the mechanisms regulating the dynamics. Several computational models have been studied which reveal to be very useful in understanding such systems. Single polymer studies give the chance of directly observing the microscopic conformations of individual chains close to equilibrium or under flow conditions, thus accessing non-equilibrium conformations.

In the case of biological filaments, their stiffness is closely related to their functions. For example, the rigidity of actin filaments is responsible for the mechanical properties of the cytoskeleton, and DNA is able to pack in the genome or inside a virus capsid thanks to its persistence length. Several works have investigated the equilibrium properties of semiflexible polymers [3,4,5,6,7,8,9,10]. The development of spectroscopic techniques and fluorescence microscopy provided insight into their non-equilibrium properties (for reviews see, e.g., Refs. [11,12]). Theoretical [13,14,15,16,17] and computational [18,19,20,21,22,23,24,25,26,27,28,29] studies helped in revealing and understanding novel dynamical, conformational, and rheological properties.

Among others, the worm-like chain model [30] proved to be accurate to describe the mechanical response of semiflexible polymers under specific conditions. Indeed, the main limitations of this model come from neglecting excluded volume effects and self-interactions between different polymer parts. The former are relevant, especially in two dimensions leading, for example, to the segregation of polymers [31]. The latter interactions, that are not relevant for strong applied fields or far from the folding temperature, are crucial for semiflexible chains with monomer–monomer interactions, such as poly(ethylene oxide) (PEO), DNA [32], or RNA [33] in poor-solvent condition [34]. Short-range attractive interactions lead to a large variety of conformations due to the competition of polymer stretching and collapse [35]. Previous experimental [36,37], theoretical, and numerical [38,39,40,41,42,43,44,45,46] studies have found that the mechanical response of self-interacting semiflexible polymers to an external stretching is very complicated. These investigations considered either chains with one of its ends grafted and the other one pulled by a force, or chains with both ends pulled away in opposite directions.

A large majority of studies has been performed in three dimensions but addressing the comprehension of stretched self-interacting polymers in two dimensions is also interesting for two main reasons: excluded-volume effects are relevant and hydrodynamic interactions can be neglected in the case of polymers strongly adsorbed on surfaces since the overall dynamics is dominated by the polymer–substrate interaction [47]. Two-dimensional realizations of these systems can, for example, be provided by DNA strongly adsorbed on a surface with one grafted end. Under these conditions the stretching of biopolymers is observed in systems with separation of biomolecules via nanochannels [48,49]. The effects of a uniform force field on two-dimensional semiflexible polymers have been considered both in experimental [47] and numerical [50,51] studies but neglecting self-attractions among monomers.

So far, a systematic study of polymers under poor-solvent condition in an external field is lacking. In the present work, the dynamical and conformational properties of a semiflexible filament, tethered by one of its ends and subjected to an external force field in two spatial dimensions, are numerically investigated. The polymer is modeled as a self-avoiding worm-like chain with self-attraction among beads. Hydrodynamics is neglected since it is assumed that local polymer friction is uniquely fixed by its interaction with the adsorbing surface. For this reason the polymer is taken to be in contact with a Brownian heat bath. This is implemented by adopting the Brownian version [52] of the multi-particle collision dynamics [53,54]. By varying stiffness and self-interaction strength, different equilibrium conformations are found. For strong mutual attraction and relative low stiffness, the structure is compact. Increasing the chain rigidity promotes the formations of folded strands. The mechanical response of the polymer to the applied force depends on the equilibrium structure. At small rigidities the initial close-packed chain is continuously unwound by the external force field. The polymer shows bistable conformations before being completely elongated. When double-stranded chains form, a “first-order”-like phase transition to the open conformation is observed in the force-extension curve. Polymer configurations are characterized by considering the gyration tensor: it is found that the relative deformation with respect to the equilibrium case along the direction normal to the force, decays as the inverse of the applied force.

The numerical model for the polymer and the Brownian heat bath are illustrated in Section 2. The results for the equilibrium conformations and the dynamic behavior are reported in Section 3. Finally, in Section 4 the main findings of this study are discussed drawing some conclusions.

## 2. Model and Method

A linear chain of length *L*, made of N+1 beads of mass *M*, is considered in two spatial dimensions. Internal forces acting on beads are due to a potential which accounts for different contributions. Connected beads interact via the harmonic potential

(1) $$U_{bond}=\frac{\kappa_{h}}{2}\sum_{i=1}^{N}\left(\right|r_{i+1}-r_{i}{\left|-r_{0}\right)}^{2},$$

where $r_{i}=\left(x_{i},y_{i}\right)$ denotes the position vector of the *i*th bead (i=1,…,N+1), $r_{0}$ is the average bond length, and the elasticity is controlled by $\kappa_{h}$. The parameter $\kappa_{h}$ is chosen in order to preserve on the average the total contour length $L=Nr_{0}$. Chain stiffness of the polymer is introduced via the bending potential

(2) $$U_{bend}=\kappa \sum_{i=1}^{N-1}\left(1-\cos\varphi_{i}\right)$$

where κ is the bending rigidity and $\varphi_{i}$ is the angle between two consecutive bond vectors. Non-bonded pair interactions are modeled by the Lennard–Jones potential

(3) $$U_{LJ}=4\epsilon \sum_{i=1}^{N-1}\sum_{j=i+2}^{N+1}\left[{\left(\frac{\sigma }{r_{i,j}}\right)}^{12}-{\left(\frac{\sigma }{r_{i,j}}\right)}^{6}\right],$$

where $r_{i,j}$ is the distance between two non-consecutive beads. A strongly attractive regime corresponds to energies $\epsilon >k_{B}T$, which determine compact structures. In the opposite limit $\epsilon <k_{B}T$ of weak self-attraction, swollen chain configurations can be observed. Here $k_{B}T$ is the thermal energy, *T* is the temperature, and $k_{B}$ is Boltzmann’s constant. The parameters κ and ϵ are varied in the present study, keeping fixed the temperature, to obtain different equilibrium conformations as later shown. In the following, for the sake of clarity, chain stiffness is characterized in terms of the length $L_{p}=2\kappa r_{0}/k_{B}T$. In the worm-like chain limit, when the Lennard–Jones potential $U_{LJ}$ is negligible, this length corresponds to the polymer persistence length [55]. However, in the present model this is not strictly true due to the coexistence of different length and energy scales [56]. Finally, in order to consider external stretching of the chain, a constant force *F* acts on every bead of the polymer. This force is directed along the *x*-direction of the Cartesian reference frame and corresponds to an external potential given by

(4) $$U_{ext}=-F\sum_{i=1}^{N+1}x_{i}.$$

The external field could be a gravitational or uniform flow field. Newton’s equations of motion of beads are integrated by the velocity-Verlet algorithm with time step $\Delta t_{p}$ [57,58].

The chain is coupled to a Brownian heat bath which is implemented by using the Brownian multi-particle collision (B-MPC) method [52,54,59] without taking into account hydrodynamic interactions. Here, we adopt the computationally efficient version proposed in Ref. [52]. In this algorithm, every bead undergoes stochastic collisions with a virtual particle of mass *M* to simulate the interaction with a fluid volume surrounding the bead. The momenta of such phantom particles are Maxwell–Boltzmann distributed with variance $Mk_{B}T$ and zero mean. The collision process is implemented via the stochastic rotation dynamics of the MPC method [54,60,61]. This corresponds to randomly rotate the relative velocity of a polymer bead, with respect to the center-of-mass velocity of the bead and its related phantom particle, by angles ±α. Collisions occur at time intervals Δt being $\Delta t>\Delta t_{p}$.

Simulations are performed with the choices $\alpha =130^{o}$, $\Delta t=0.1t_{u}$, with time unit $t_{u}=\sqrt{mr_{0}^{2}/\left(k_{B}T\right)}$, M=5m, $\kappa_{h}r_{0}^{2}/\left(k_{B}T\right)=10^{4}$, $\sigma =r_{0}$, N=50, and $\Delta t_{p}=10^{-2}\Delta t$. The value of $\kappa_{h}$ ensures that the polymer length *L* is constant within 1% for all systems.

## 3. Numerical Results

Polymers are initialized with beads randomly aligned along the *x*-direction and allowed to equilibrate. The position $r_{1}$ of the first bead is fixed at the origin (0,0) of the Cartesian reference frame while no orientation is enforced for the first bond. When taking into account the action of the uniform force field, simulations are started from the equilibrium configurations of chains and run until reaching steady states during which average quantities are computed. We consider semiflexible polymers with values of the bending rigidity κ, such that $0.1\leq L_{p}/L\leq 2$, and interaction energies $\epsilon /k_{B}T=0.25,2$.

### 3.1. Equilibrium Polymer Conformations

**Figure 1:**
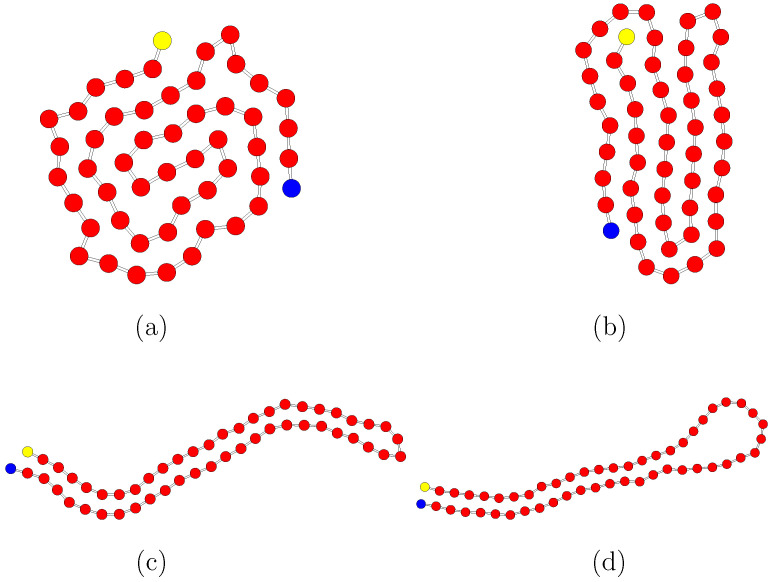
Equilibrium polymer conformations for $L_{p}/L=0.1$ (**a**), 0.2 (**b**), 0.4 (**c**), 0.8 (**d**) with $\epsilon /(k_{B}T)=2$. Blue and yellow beads denote the first and last ones, respectively. Polymer beads and bonds are not in scale to allow a better visualization.

In this Section, the equilibrium properties of polymers are obtained and characterized by varying the bending rigidity and the interaction energy. When considering the value $\epsilon /k_{B}T=0.25$, non-bonded interactions are negligibly small and the model corresponds to the worm-like chain model [30], as shown in the following. In this case, the filament assumes a swollen configuration with spatial correlations, in the direction of the chain tangent, on a length scale given by the persistence length. A different scenario occurs when non-bonded interactions become relevant. Equilibrium configurations for $\epsilon /k_{B}T=2$ and different values of the length $L_{p}$ are shown in Figure 1.

For the smallest value $L_{p}/L=0.1$ (Figure 1a) the chain has a globule structure which is very compact. Increasing the stiffness promotes the formation of folded bundles. A configuration with five rod-like strands is shown in Figure 1b for $L_{p}/L=0.2$. The energy penalty, which is proportional to (1−cosφ) and increases with the bending angle φ at turning points, is compensated by the energy gain from bead–bead attractions. The number of strands diminishes when increasing κ. A structure formed by two facing strands is observed at $L_{p}/L=0.4$ (Figure 1c). For this value of $L_{p}$, the average bending energy diminishes since the number of turning points reduces, and the average Lennard–Jones energy increases. A further increase in chain stiffness induces the formation of hairpin conformations (Figure 1d for $L_{p}/L=0.8$). This causes a second rise in the bending energy whose energetic penalty can still be compensated by the mutual attraction between monomers. Finally, at $L_{p}≃L$ the polymer cannot sustain any closed configuration and a rod-like structure is observed for values $L_{p}≳L$. In this latter range, the average value of $U_{LJ}$ exhibits a sharp increase while the average bending energy decreases.

**Figure 2:**
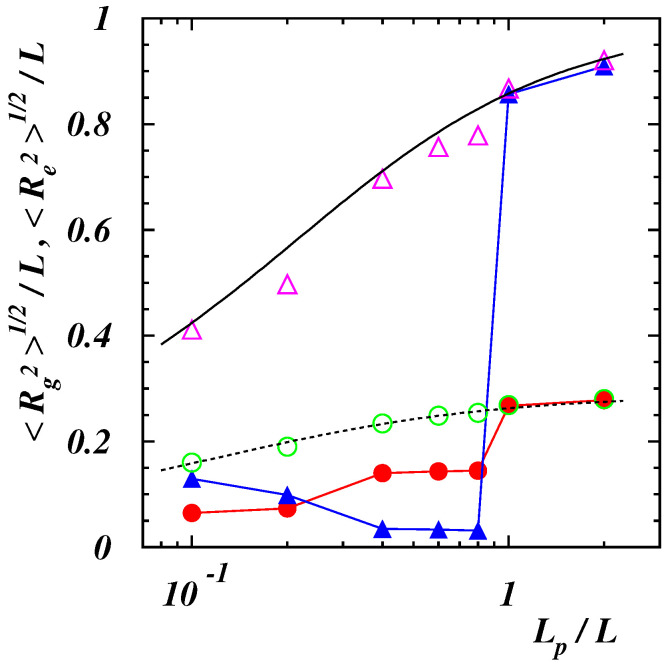
Root -mean-square end-to-end distance $R_{e}$ of a polymer in absence of an external force for $\epsilon /(k_{B}T)=0.25$ (purple open triangles), 2 (blue filled triangles), and gyration radius $R_{g}$ for $\epsilon /(k_{B}T)=0.25$ (green open circles), 2 (red filled circles). The full and dashed black lines correspond to the analytical predictions (6) and (7), respectively, in the case of continuous semiflexible polymers [55].

In order to characterize the conformations of chains, it is useful to consider the root-mean-square values of the end-to-end distance ${\langle R_{e}^{2}\rangle }^{1/2}$, where $R_{e}=\left|r_{N+1}-r_{1}\right|$, and of the gyration radius ${\langle R_{g}^{2}\rangle }^{1/2}$. By computing the gyration tensor

(5) $$G_{\alpha \beta }=\frac{1}{N+1}\sum_{i=1}^{N+1}\Delta r_{i,\alpha }\Delta r_{i,\beta },$$

where $\Delta r_{i,\alpha }$ is the position of the *i*-th monomer in the center-of-mass reference frame of the chain and the Greek index denotes the Cartesian component, the gyration radius can be obtained as $R_{g}^{2}=\sum_{\alpha }G_{\alpha \alpha }$. The computed values of ${\langle R_{e}^{2}\rangle }^{1/2}$ and ${\langle R_{g}^{2}\rangle }^{1/2}$ for the two values of ϵ as functions of the dimensionless length $L_{p}/L$ are presented in Figure 2.

For the smallest value $\epsilon /k_{B}T=0.25$ the numerical results show a quantitative agreement with the theoretical predictions for a continuous semiflexible chain [55]

(6) $$\begin{array}{ccc} \langle R_{e}^{2}\rangle & = & 2L_{p}L\left[1-\frac{L_{p}}{L}\left(1-e^{-L/L_{p}}\right)\right], \end{array}$$

(7) $$\begin{array}{ccc} \langle R_{g}^{2}\rangle & = & L_{p}L\left[\frac{1}{3}-\frac{L_{p}}{L}+2{\left(\frac{L_{p}}{L}\right)}^{2}-2{\left(\frac{L_{p}}{L}\right)}^{3}\left(1-e^{-L/L_{p}}\right)\right]. \end{array}$$

This confirms that the self-interaction energy is negligible for this choice of ϵ and the polymer behaves as a worm-like chain. The behavior is different for the highest value of the energy ϵ. The end-to-end distance is smaller than in the previous case and decreases to reach its minimum value when the chain consists of two strands folded on each other ($0.4≲L_{p}/L≲0.8$). Then, ${\langle R_{e}^{2}\rangle }^{1/2}$ jumps to values comparable to those of semiflexible polymers at $L_{p}/L≃1$. The average gyration radius ${\langle R_{g}^{2}\rangle }^{1/2}$ is at a minimum when compact conformations are observed ($L_{p}/L≲0.2$), then increases to a value which remains constant as long as the chain consists of two strands, and finally reaches the equilibrium values of worm-like chains when the polymer assumes a rod-like structure.

**Figure 3:**
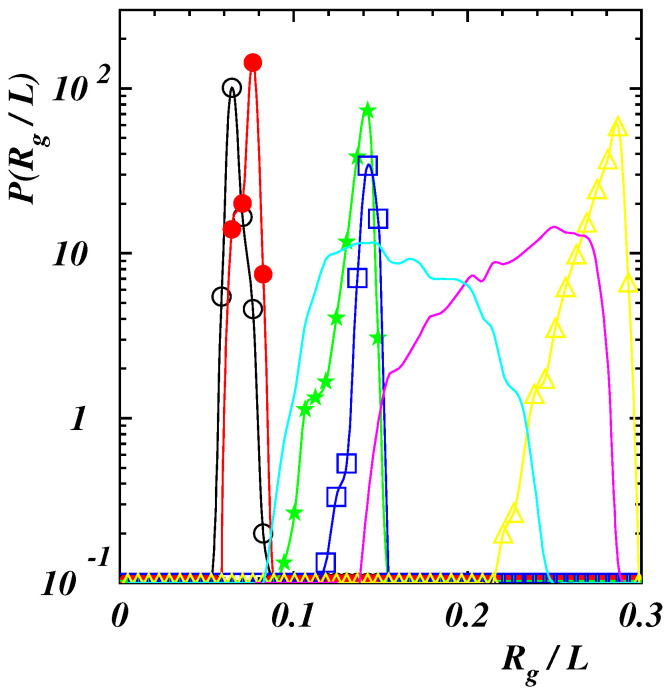
Normalized probability distribution function of the gyration radius $R_{g}$ in absence of the external force for $L_{p}/L=0.1$ (black open circles), 0.2 (red filled circles), 0.4 (green filled stars), 0.8 (blue open squares), 2 (yellow open triangles) with $\epsilon /(k_{B}T)=2$, and for $L_{p}/L=0.1$ (cyan line), 0.4 (purple line) with $\epsilon /(k_{B}T)=0.25$.

Normalized probability distribution functions (PDFs) of the polymer gyration radius $R_{g}$ are depicted in Figure 3 under equilibrium conditions.

For the highest value of the interaction energy ϵ, when the polymer is compact, curves are very narrow corresponding to the fact that the chain global conformation does not change significantly in time. The two curves with $L_{p}/L=0.1,0.2$ almost overlap with peaks located at $R_{g}/L≃0.07$. When considering double-stranded chains, the curve at $L_{p}/L=0.4$ is broader since the chain fluctuates along its length. The peak is at $R_{g}/L≃0.14$ as in the case with $L_{p}/L=0.8$ where the PDF is narrower since the structure is quite rigid. Finally, when the polymer assumes a rod-like conformation ($L_{p}/L=2$), the position of the PDF peak moves to $R_{g}/L≃0.28$. For a comparison two PDFs in the case of weak self-attraction ($\epsilon /k_{B}T=0.25$) are also presented in the figure. Curves are broader than in the previous case due to the fact that chains are more prone to fluctuate since the mutual attraction is negligible. The peaks are located at larger values of $R_{g}$ with respect to the case with $\epsilon /k_{B}T=2$, for the same stiffness, corresponding to more elongated structures.

### 3.2. Polymer Stretching in Uniform Force Field

When the polymer is subject to the external force, it is stretched along the direction of the force. In order to characterize the elongation of the chain, the average deficit length-ratio $\delta =1-\langle x_{N+1}\rangle /L$ as a function of the applied force is considered. $\langle x_{N+1}\rangle$ is the average extension of the chain along the force direction computed as the average value of the *x*-component of the end-to-end vector $R_{e}=r_{N+1}-r_{1}$. When self-attraction is negligible, in the limit $|x_{N+1}|\to L$ it results [47,62]

(8) $$\delta ∼{\left(\frac{1}{F_{2}}\right)}^{1/2}$$

with $F_{2}=NFL_{p}/\left(k_{B}T\right)$. For quite strong force fields or very small bending rigidities the behavior does not depend on the stiffness and is given by [38,50,63]

(9) $$\delta ∼\frac{1}{F_{1}}$$

where $F_{1}=NFr_{0}/\left(k_{B}T\right)$, as for flexible chains [64].

**Figure 4:**
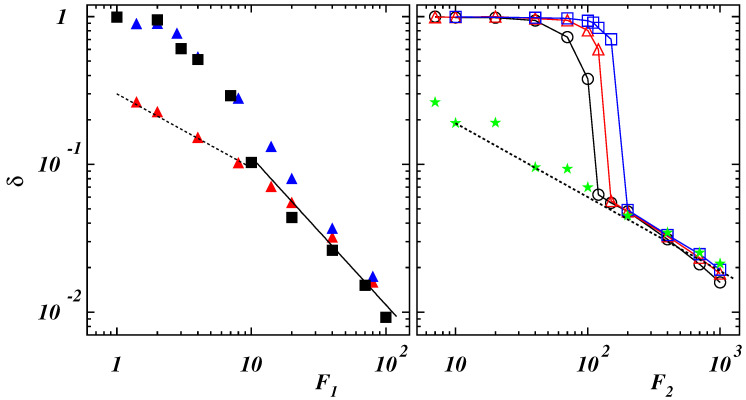
(**Left** panel) Mean deficit length-ratio along the direction of the external force as a function of the dimensionless force $F_{1}=NFr_{0}/\left(k_{B}T\right)$ for $L_{p}/L=0.1$ (blue filled triangles), 0.2 (black filled squares) with $\epsilon /(k_{B}T)=2$, and for $L_{p}/L=0.1$ (red filled triangles) with $\epsilon /(k_{B}T)=0.25$. The dashed and full lines have slopes −1/2 and −1, respectively. (**Right** panel) Mean deficit length-ratio along the direction of the external force as a function of the dimensionless force $F_{2}=NFL_{p}/\left(k_{B}T\right)$ for $L_{p}/L=0.4$ (black open circles), 0.6 (red open triangles), 0.8 (blue open squares), 2 (green filled stars) with $\epsilon /(k_{B}T)=2$. The dashed line has slope −1/2.

Different behaviors can be expected for self-interacting semiflexible polymers. When the filament is pulled at one end by a constant force, a sharp transition appears in the force vs. elongation curves [38] whose sharpness is enhanced by bending rigidity [42]. Simulations results of the present model are illustrated in Figure 4 as functions of applied force for different values of the ratio $L_{p}/L$.

**Figure 5:**
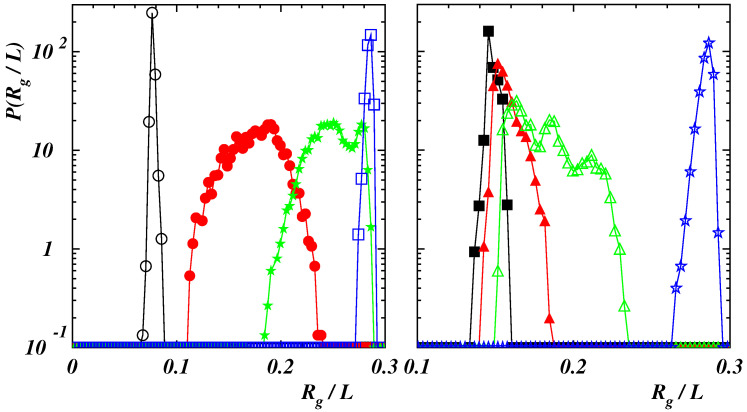
(**Left** panel) Normalized probability distribution function of the gyration radius $R_{g}$ for $F_{1}=NFr_{0}/\left(k_{B}T\right)=1$ (black open circles), 4 (red filled circles), 7 (green filled stars), 20 (blue open squares) with $L_{p}/L=0.2$ and $\epsilon /(k_{B}T)=2$. (**Right** panel) Normalized probability distribution function of the gyration radius $R_{g}$ for $F_{2}=NFL_{p}/\left(k_{B}T\right)=100$ (black filled squares), 120 (red filled triangles), 150 (green open triangles), 200 (blue filled stars) with $L_{p}/L=0.8$ and $\epsilon /(k_{B}T)=2$.

In case of $L_{p}/L\leq 0.2$, corresponding to compact initial states (see Figure 1), data collapse is obtained when plotting values of δ as functions of the dimensionless force $F_{1}=NFr_{0}/\left(k_{B}T\right)$ (left panel of Figure 4). The initial structure is tilted in the direction of the force and only slightly deformed as long as $F_{1}≲1$. This can also be appreciated when considering the normalized PDFs of the gyration radius: in the case with $L_{p}/L=0.2$ and $F_{1}=1$, the PDF exhibits a narrow peak (see Figure 5 (left panel)).

By increasing the force, the extension increases smoothly since the globule is partially unwound, similarly to what holds for single-stranded DNA and RNA [38,39]. A chain-and-blob [65] configuration can be observed where the blob at the end fluctuates in shape and size due to thermal fluctuations (see the Supplementary Video S1 for $L_{p}/L=0.2$ and $F_{1}=4$). The corresponding PDF broadens while still displaying a single peak which moves toward larger values of $R_{g}$. At $F_{1}≃7$, the chain is stretched although, from time to time, the final part can be still folded due to self-attraction (see the Supplementary Video S2 for $L_{p}/L=0.2$ and $F_{1}=7$). The PDF of $R_{g}$ exhibits two peaks corresponding to fully elongated and partially bent conformational states which are stable for relatively long times to be clearly observed. This multi-peak feature is similar to that observed for pulled semiflexible polymers under poor-solvent condition [42,66,67] and proteins subject to a uniform flow [68]. By further increasing the force, the polymer is completely elongated with a narrow PDF of $R_{g}$ whose position shifts continuously to larger values of $R_{g}$. The relation (9), observed once $F_{1}≳10$, indicates that the chain behaves as a semiflexible polymer under strong force. As a matter of comparison, we report also the results in the case when self-attraction is negligible for a similar bending rigidity (see the data for $L_{p}/L=0.1$ and $\epsilon /(k_{B}T)=0.25$ in the left panel of Figure 4). The behavior at small force values is different with the deficit length-ratio decaying as $F^{-1/2}$, which is typical of semiflexible polymers without self-interaction. By increasing the force, the dependence (9) is recovered with the values of δ collapsing onto the ones for $\epsilon /(k_{B}T)=2$.

**Figure 6:**
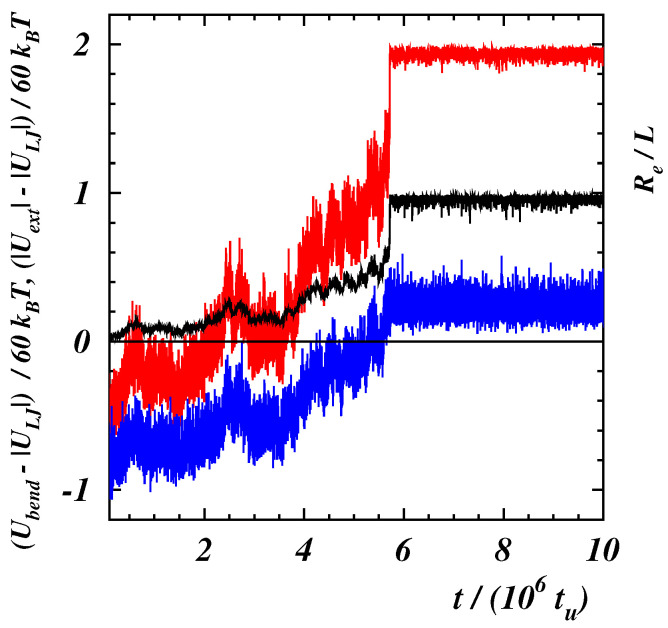
Potential energy differences $U_{bend}-\left|U_{LJ}\right|$ (blue line) and $|U_{ext}\left|-\right|U_{LJ}|$ (red line) as functions of time in the case of the polymer with $L_{p}/L=0.8$ and $\epsilon /(k_{B}T)=2$ for $F_{2}=NFL_{p}/\left(k_{B}T\right)=200$. The time behavior of the end-to-end distance $R_{e}$ is also shown (black line).

When the stiffness of the chain is such that a polymer exhibits a double-stranded conformation, the mechanical response to the external force is different as it can be seen in the right panel of Figure 4 where the average deficit length-ratio δ is plotted as a function of the dimensionless force $F_{2}=NFL_{p}/\left(k_{B}T\right)$. Three regimes can be distinguished. For values $F_{2}≲10$ the two strands are aligned along the force direction but there is no relative motion of the last bead with respect to the first one, kept fixed in the origin, so that $\langle x_{N+1}\rangle ≃0$. In this case, the PDF of the gyration radius is narrow (see the curve corresponding to the case with $L_{p}/L=0.8$ and $F_{2}=100$ in the right panel of Figure 5). When the force is increased, the strand, which is not constrained to the origin, moves over the other part. This causes a broadening of the PDF (see the curve with $F_{2}=120$). A stronger force facilitates a larger sliding. Due to the overall fluctuations of the polymer, the final bead does not attain a fixed position relative to the first bead but can move back and forth along the chain (see the Supplementary Video S3 for $L_{p}/L=0.8$ and $F_{2}=150$). The PDF is characterized by more peaks corresponding to different stable configurations assumed by the polymer in the same ensemble. However, the final part of the chain cannot slide continuously due to the finite rigidity so that larger forces are required to unfold the polymer. The time behavior of the energy terms $\left(U_{bend}-|U_{LJ}|\right)$ and $\left(|U_{ext}|-|U_{LJ}|\right)$ is shown in Figure 6 in the case with $L_{p}/L=0.8$ for $F_{2}=200$.

The last bead can slide when it occurs that $|U_{ext}\left|>\right|U_{LJ}|$, as witnessed by the increase of the end-to-end distance $R_{e}$ also reported in the figure. As $R_{e}$ becomes continuously larger, $U_{bend}$ approaches $|U_{LJ}|$ and, when $U_{bend}$ exceeds $|U_{LJ}|$, the polymer swells abruptly signaling a “first-order”-like phase transition. (see the Supplementary Video S4 for $L_{p}/L=0.8$ and $F_{2}=200$). Once the polymer is completely elongated, the PDF has again a single peak whose position jumps discontinuously to a larger value. The force required to unzip completely the polymer increases with the bending rigidity and the transition from the folded state to the elongated one becomes sharper, as in the case of the unzipping of double-stranded DNA [39,45,69]. When the polymer is completely unfolded, the values of δ for different bending rigidities lay on the same curve following the decay (8) of semiflexible filaments, as it happens in the case of the stiffer chain with $L_{p}/L=2$.

**Figure 7:**
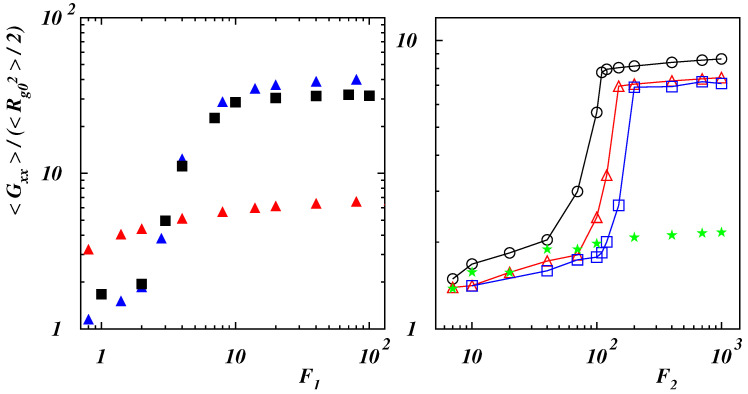
(**Left** panel) Radius of gyration tensor component along the force direction with respect to the equilibrium value as a function of the dimensionless force $F_{1}=NFr_{0}/\left(k_{B}T\right)$ for $L_{p}/L=0.1$ (blue filled triangles), 0.2 (black filled squares) with $\epsilon /(k_{B}T)=2$, and for $L_{p}/L=0.1$ (red filled triangles) with $\epsilon /(k_{B}T)=0.25$. (**Right** panel) Radius of gyration tensor component along the force direction with respect to the equilibrium value as a function of the dimensionless force $F_{2}=NFL_{p}/\left(k_{B}T\right)$ for $L_{p}/L=0.4$ (black open circles), 0.6 (red open triangles), 0.8 (blue open squares), and 2 (green filled stars) with $\epsilon /(k_{B}T)=2$.

**Figure 8:**
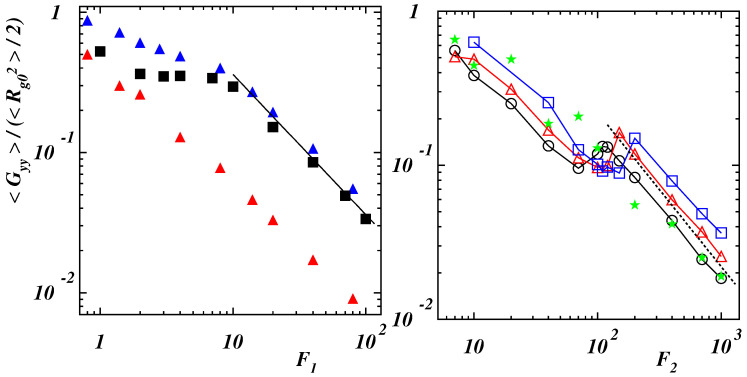
(**Left** panel) Radius of gyration tensor component along the *y*-direction with respect to the equilibrium value as a function of the dimensionless force $F_{1}=NFr_{0}/\left(k_{B}T\right)$ for $L_{p}/L=0.1$ (blue filled triangles), 0.2 (black filled squares) with $\epsilon /(k_{B}T)=2$, and for $L_{p}/L=0.1$ (red filled triangles) with $\epsilon /(k_{B}T)=0.25$. The full line has slope −1. (**Right** panel) Radius of gyration tensor component along the *y*-direction with respect to the equilibrium value as a function of the dimensionless force $F_{2}=NFL_{p}/\left(k_{B}T\right)$ for $L_{p}/L=0.4$ (black open circles), 0.6 (red open triangles), 0.8 (blue open squares), and 2 (green filled stars) with $\epsilon /(k_{B}T)=2$. The dashed line has slope −1.

Polymer deformation can be characterized in terms of the gyration tensor (5). The ratios $\langle G_{\alpha \alpha }\rangle /\left(\langle R_{g0}^{2}\rangle /2\right)$ (α∈{x,y}) are presented in Figure 7 and Figure 8, where $\langle R_{g0}^{2}\rangle$ is the mean-square value of the gyration radius calculated at equilibrium. For values of the bending rigidity $L_{p}/L\leq 0.2$ (see the left panel of Figure 7), the behavior is similar and the polymer is smoothly deformed in the force direction as long as the blob is unwound ($1≲F_{1}≲10$).

Once the chain has been disentangled ($F_{1}>10$), the deformation reaches a value which does not change significantly with the force. Due to the initial compact structure, the ratio of deformation is considerably larger with respect to the case with negligible self-interaction which is also shown in the left panel of Figure 7 for $L_{p}/L=0.1$. In the right panel of the same figure, the deformation is shown as a function of the dimensionless force $F_{2}$ when the polymer has a double-stranded initial configuration. Initially, in case of $\langle x_{N+1}\rangle ≃0$, the force slightly elongates the chain with respect to the equilibrium case. As soon as the last bead starts to slide over the filament, the deformation increases rapidly with steepness depending on the bending rigidity. Finally, it reaches a constant value when the polymer is fully elongated along the force direction. The smaller relative deformation corresponds to the more stiff polymer whose initial configuration is a stiff hairpin (see Figure 1d). When the bending rigidity is such that no closed structure can form ($L_{p}/L=2$), the chain is smoothly elongated over the whole range of explored forces with a final value sensibly smaller than the one corresponding to initially double-stranded chains.

Along the *y*-direction, normal to the force, the relative deformation diminishes as a function of the dimensionless force $F_{1}$ when $L_{p}/L\leq 0.2$ (left panel of Figure 8).

As long as the chain maintains a compact structure, the decrease is weak while it becomes steeper when the polymer is open under the action of the external driving. At values $F_{1}>10$, data collapse and a power-law with dependence $F_{1}^{-1}$ can be observed. When self-interaction is negligible, the behavior is similar but the deformation is much smaller due to the lack of a compact-like initial structure. More interesting appears to be what happens for the range of stiffness corresponding to double-stranded conformations. The initial values of $\langle G_{yy}\rangle /\left(\langle R_{g0}^{2}\rangle /2\right)$ decrease, due to the stretching of the two strands, with a similar trend. When the folded strands open, an overshoot can be observed that is due to the larger fluctuations of the chain. The deformation then follows a power-law decay with dependence $F_{2}^{-1}$. The data for the initially stretched polymer ($L_{p}/L=2$) show a similar behavior without the aforementioned overshoot.

## 4. Discussion and Conclusions

The dynamical and conformational properties of semiflexible polymers under poor-solvent condition in a uniform force field have been numerically studied. The chain has been anchored at one end, confined in two dimensions and placed in contact with a Brownian heat bath implemented by the stochastic version of the multi-particle collision dynamics.

The equilibrium conformation depends both on the stiffness and on the self-interaction strength. When the self-attraction energy is smaller compared to the thermal energy, the chain behaves as a semiflexible filament. In the opposite limit of strong mutual attraction, different configurations are obtained. At low bending rigidity the polymer assumes a compact structure. By increasing the stiffness, patterns of folded bundles emerge where the number of strands reduces as the chain becomes more rigid. A larger number of polymer beads, with respect to the value here considered, would promote folded conformations with more strands as observed for three-dimensional semiflexible polymers [70]. Finally, rod-like conformations are recovered for high values of the rigidity.

The mechanical response to the action of the external force depends on the initial equilibrium structure. For small bending rigidity the compact structure is continuously unwound and stretched by the force. On the other hand, when the polymer consists of two facing strands, a “first-order”-like phase transition is observed from the folded to the stiff conformation. These behaviors are highlighted in the force-extension relations, as well as at the probability distribution functions of the gyration radius. The deformation of the radius of gyration with respect to the equilibrium value along the direction normal to the force is found to decay as the inverse of the applied force.

Although hydrodynamics interactions have been neglected in this investigation, it is known that such interactions are not essential in the case of semiflexible polymers since only logarithmic corrections are expected [5]. Therefore, the present results also describe the behavior of a self-attractive semiflexible polymer placed in a uniform flow field as long as the chain follows the fluid flow. We hope that this study will stimulate theoretical studies and experimental investigations to confirm the outlined phenomenology.

## Data Availability

Data are available upon reasonable request.

## Supplementary Materials

The following supporting information can be downloaded at: https://www.mdpi.com/article/10.3390/polym14214762/s1, Video S1: Animation of polymer stretching for $L_{p}/L=0.2$ and $\epsilon /(k_{B}T)=2$ with $F_{1}=4$; Video S2: Animation of polymer stretching for $L_{p}/L=0.2$ and $\epsilon /(k_{B}T)=2$ with $F_{1}=7$; Video S3: Animation of polymer stretching for $L_{p}/L=0.8$ and $\epsilon /(k_{B}T)=2$ with $F_{2}=150$; Video S4: Animation of polymer stretching for $L_{p}/L=0.28$ and $\epsilon /(k_{B}T)=2$ with $F_{2}=200$.
